# Sex differences in neuronal systems function and behaviour: beyond a single diagnosis in autism spectrum disorders

**DOI:** 10.1038/s41398-021-01757-1

**Published:** 2021-12-09

**Authors:** Olivia O. F. Williams, Madeleine Coppolino, Melissa L. Perreault

**Affiliations:** Department of Biomedical Sciences, University of Guelph, Guelph, ON Canada

**Keywords:** Molecular neuroscience, Neuroscience, Diagnostic markers, Predictive markers

## Abstract

Autism spectrum disorder (ASD) is a complex neurodevelopmental disorder that is associated with functional brain alterations that underlie the expression of behaviour. Males are diagnosed up to four times more than females, and sex differences have been identified in memory, cognitive flexibility, verbal fluency, and social communication. Unfortunately, there exists a lack of information on the sex-dependent mechanisms of ASD, as well as biological markers to distinguish sex-specific symptoms in ASD. This can often result in a standardized diagnosis for individuals across the spectrum, despite significant differences in the various ASD subtypes. Alterations in neuronal connectivity and oscillatory activity, such as is observed in ASD, are highly coupled to behavioural states. Yet, despite the well-identified sexual dimorphisms that exist in ASD, these functional patterns have rarely been analyzed in the context of sex differences or symptomology. This review summarizes alterations in neuronal oscillatory function in ASD, discusses the age, region, symptom and sex-specific differences that are currently observed across the spectrum, and potential targets for regulating neuronal oscillatory activity in ASD. The need to identify sex-specific biomarkers, in order to facilitate specific diagnostic criteria and allow for more targeted therapeutic approaches for ASD will also be discussed.

## Introduction

Autism spectrum disorders (ASD) are a complex group of neurodevelopmental disorders characterized by a broad range of cognitive and behavioural symptoms of varying severity. With an increasing worldwide prevalence ASD affects approximately 1 in 160 children [1], and although genetic factors contributing to ASD have been identified, the disorder is most often idiopathic with no known cause [2]. ASD is primarily diagnosed during childhood or early adolescence when social communication difficulties are present alongside impaired social interactions, difficulty understanding others’ emotions, excessively repetitive behaviours, restricted interests, insistence on sameness, and in 90% of cases sensory atypicalities are observed [3–5]. It is also important to note that the type of sensory atypicalities vary between individuals diagnosed upon the spectrum, as hypo- or hypersensitivities can occur [5, 6]. Unfortunately, due to the wide range of behavioural characteristics, there is frequent misdiagnosis or a delayed diagnosis [7]. Added to the complexity of ASD is the high comorbidity with other neurological conditions including attention deficit hyperactivity disorder, intellectual disability, anxiety, obsessive-compulsive disorder, seizures and cerebral palsy [8, 9]. Although pharmacological interventions are often used to treat the comorbidities, currently there is no cure for ASD, with behavioural therapies most often employed.

There are prominent sex differences in both the prevalence and behavioural characteristics of ASD. Male individuals are up to four times more likely than female individuals to develop ASD and are most often diagnosed in childhood, whereas females are commonly diagnosed later in adolescence, or even in early adulthood [10]. For many years it was assumed that male and female patients exhibited similar symptoms on the spectrum, as reports detailed a “hyper-masculinization” of many of the behavioural traits expressed in individuals with ASD, which consequently resulted in the “Extreme Male Brain” (EMB) theory [11–13]. The EMB theory is an extension of the “Empathizing-Systemizing” (E-S) theory of sex differences, as it infers that the cognitive profile of individuals with ASD is similar to an extreme version of that of a neurotypical male [11]. More specifically, the E-S theory aims to explain sex differences in populations, independent of age or an ASD diagnosis, by categorizing all individuals into five groups depending on their expression of empathy and systemizing [14]. Empathizing is the ability to sense others emotions, usually allowing feelings to guide appropriate responses, whereas systemizing is the ability to take in information and create rules based on this information, allowing one to expect an outcome and separate emotions from day-to-day tasks [11]. In general, neurotypical males and females express different cognitive profiles with females appearing to have a greater ability to empathize, and males exhibiting stronger systemizing abilities [11]. The EMB theory of ASD proposes that these individuals express a more extreme phenotype of heightened systemizing over empathizing which relates to the “hyper-masculinization” of some behavioural traits in the disorder [11]. The mechanisms underlying such an extreme male profile are not fully understood, though female individuals with ASD have shown significantly higher testosterone levels compared to neurotypical females, which potentially underlies this theory [15]. Despite females expressing such characteristics as described by the EMB theory, it is important to emphasize that not all female individuals with ASD exhibit all male-specific characteristics [12] and therefore the EMB theory of ASD remains controversial [16].

Sexual dimorphisms in ASD symptoms and symptom severity exist in memory, cognitive flexibility, verbal fluency, and social communication [10, 17–19]. For example, male individuals express difficulties with social communication/interaction skills and greater repetitive and stereotyped behaviours [10, 17–19], whereas females exhibit reduced verbal control, increased fine motor skills, have more difficulty internalizing and expressing emotions, but are more sociable [10, 17]. As the presentation of behaviours in ASD differs across the spectrum, where sex differences are of critical importance, it is highly probable that the associated underlying pathologies differ. Despite little being known about sex differences in the molecular and cellular mechanisms that contribute to ASD, it has been suggested that more drastic neurological changes in neuronal signalling are required for ASD progression in females [20, 21]. However, the fact that children are most often grouped in clinical research studies, rather than separated into sub-groups based on symptoms along the spectrum and sex, has likely contributed to the lack of current knowledge of sex differences. This knowledge could be used to establish much needed biomarkers and novel therapeutic approaches to improve diagnosis, prevention, and treatment of these disorders.

Sex-specific biomarkers in ASD are sorely needed if more tailored therapies are to be developed, yet the identification of biomarkers in ASD in general has proven to be elusive. Some evidence suggests that neuronal oscillatory signatures, or brain waves, may represent a viable approach as these oscillatory patterns are widely accepted to be tightly coupled to behavioural states [22–25], and to play a central role in the pathology of many neuropsychiatric and neurodevelopmental disorders including ASD [20, 25–31]. This review will therefore focus on what is presently known about oscillatory dysfunction in ASD, with an emphasis on sex differences where possible. Potential targets in the regulation of neuronal oscillatory activity in the context of ASD will also be discussed.

## Neuronal oscillations in ASD

Macroscopic oscillations, derived from the synchronous activity of neuronal ensembles, generate rhythmic brain electrophysiological patterns that are coupled to behavioural and cognitive states (i.e., concentration, attention) [32, 33]. These oscillations are a key indicator of the communication status between neurons, neuronal connectivity or synchrony, with these connections forming circuits that collectively make up the large networks that are involved in cognitive processes [25]. Measurement of these electrophysiological rhythms, or of functional network activity, can be done non-invasively in humans through magnetic resonance imaging (MRI) [18], functional MRI (fMRI) [34–36], electroencephalography (EEG) [37–39], and magnetoencephalography (MEG) [40]. The blood-oxygen-level dependent (BOLD) signal detected in fMRI is also a potential measure of neuronal oscillations as it has been shown to be linked to neuronal oscillatory activity in various brain regions including visual and auditory cortices [34, 35].

Cognitive states have been linked with the coordinated activity of oscillatory rhythms at both low and high frequencies [22, 24, 25]. Oscillatory rhythms are conventionally defined frequencies which are broken down into five distinct waveforms [22]. In adults, low-frequency bands, delta (0.5–4 Hz), theta (>4–8 Hz), and alpha (>8–12 Hz), are slow waves and are critical in long-distance or between region communication, whereas the high frequency bands, beta (>12–30 Hz), and gamma (>30 Hz) are fast waves that play a role in short-distance or within region communication [22]. However, it is noteworthy that the margins of corresponding bands appear to be lower in infants and children [41]. The synchronization of oscillations plays an important role in information transmission, with the coordinated synchronous activity of neuronal ensembles, termed coherence, integral for effective communication between them [22, 26, 42]. Coherence, a measure of functional connectivity, is often utilized to analyze neuronal activity and communication alterations in various disorders as when functional network activity and coherence begins to fail due to pathogenic alterations, cognitive disruptions can arise [28, 43].

As a neurodevelopmental disorder, neuronal connectivity alterations are observed throughout the development of children with ASD [44]. Such differences in neuronal oscillatory function and connectivity have been observed in several brain regions implicated in ASD including the frontal [18, 36, 37, 39], temporal [18, 40], and occipital cortices [18, 38]. Studies have focused on the relationship between neuronal oscillatory alterations and behaviours in ASD, although many inconsistencies exist, potentially as a result of both the lack of inclusion of sex differences in the studies, as well as the grouping of individuals into a single “ASD group” despite significant variations in symptom presentation.

### Delta

Several studies have examined delta power in ASD with conflicting results. For example, in children aged 7–13 years diagnosed with ASD, clinical EEG studies indicate that there is lower frontal cortical delta power as well as lower delta coherence across a number of regions [45, 46]. Similarly, in a prospective longitudinal EEG study of children with a sibling diagnosed with ASD, and therefore defined as having an elevated familial likelihood of developing ASD, it was shown that delta power was lower in these children at 6 months of age, although this normalized over the subsequent 18 months [47]. With these findings, it could be speculated that reduced delta power may serve as a biomarker for ASD, except it is not consistently observed, and indeed, elevated delta power has also been demonstrated. For example, an EEG study examining children aged 6–15 years diagnosed with ASD (specifically Asperger’s syndrome) showed increased delta power during non-REM sleep in frontal cortices [48], a finding also supported by an MEG study that demonstrated elevated resting-state frontal relative delta power in awake individuals with ASD [40]. In a rodent model used for the study of fragile X syndrome, whereby the fragile X mental retardation (*fmr-1)* gene was deleted in mice, there was an elevation in delta power reported within the auditory cortex and frontal cortex [49]. However, other studies show no changes in delta power in the frontal and posterior cortex of adults with a clinical diagnosis of ASD in eyes open or closed resting state [50], or in whole brain analysis of adults diagnosed specifically with fragile X syndrome [51]. Together these studies indicate that while age-specific alterations in resting frontal cortical delta power may accompany an ASD diagnosis in children, there are clear discrepancies in the findings as to whether delta power is up- or downregulated. A more clear delineation of the ASD subtypes utilized in the studies, and the sex of the participants, would provide important insights into the precise role of cortical delta power in ASD.

### Theta

In general, a role for theta in ASD has been poorly characterized, surprising given its predominant role in other disorders that display cognitive changes, such as schizophrenia [52] and attention deficit hyperactivity disorder [53]. Resting-state [46] and cognitive functioning EEG [54, 55] studies have observed a decrease in theta band coherence throughout the brain, and specifically the occipital cortex, in children with ASD. Similarly, infants with a greater familial likelihood of developing ASD have significantly lower frontal theta power than their control counterparts at 6, 12, 18 and 24 months of age [47]. In the right posterior brain, however, elevated theta power has been reported in both children [45] and adults [50, 56] with ASD. Some evidence also indicates lower theta coherence across the frontal, temporal, and parietal cortices with posterior regions of the brain in children [45, 48, 55, 57] and adults [48]. With respect to preclinical work, there is little information on theta waves using ASD model systems. Although in one study, the impact of partial deletion of the pogo transposable element derived with ZNF domain (*Pogz*), a gene with recurrent mutations in ASD, was evaluated using heterozygous loss of function mice (*Pogz*^*+/−*^) [58]. In this model, a deficit in hippocampal-prefrontal theta synchrony was demonstrated and was thought to underlie anxiety-related avoidance, a behavioural characteristic of these mice [58].

### Alpha

Unlike the delta and theta frequencies, the alpha frequency has been studied to a much greater extent in ASD. Some studies identified lower alpha power in central and posterior brain regions in individuals with ASD during sleep and resting states [48, 56, 59–62]. This suppression of alpha power was age-independent, occurring in children 4–8 years of age [60, 62], adolescents 12–18 years of age [59, 61], as well as adults 18 years and older [48, 56, 62]. In one longitudinal study, Walsh and colleagues [62] also identified lower frontal alpha power in anesthetized children (mean 5.4 years of age), which continued to decline as they approached early adulthood (mean 22.5 years of age). In temporal, parietal and occipital regions, reports have also shown an elevation in alpha power in children, adolescents, and adults with ASD [40, 50, 60, 63, 64]. Alterations in alpha power for individuals with an elevated familial likelihood of ASD have also been identified [47, 65]. Gabard-Durnam and colleagues [65] identified changes in frontal alpha power over a 6-month period within the first two years of life, with infants with increased ASD likelihood displaying a shift in increased frontal alpha power from left to right hemispheres, while children without this likelihood displaying a progression from right to left. In another study, infants that had an increased chance of developing ASD exhibited lower resting frontal alpha power that increased as the child aged from 6- to 24-months [47]. It was postulated that such early disruptions in alpha power may potentially predict ASD outcome later in life [47]. A reduction in alpha coherence was also shown in adolescents [57] and adults [48, 50, 56] with ASD between frontal, fronto-temporal, parieto-occipital and posterior regions, indicating disruptions in inter-regional communication at this frequency. Further, the finding that an increase in temporal and parietal alpha power was significantly correlated with greater atypicalities in social behaviour, suggests that varying levels of alpha power may be correlated to the various symptoms of ASD [40]. The researchers correlated alpha power in the anterior temporal cortex and parietal cortex to social responsiveness scale (SRS) scores, where greater SRS scores were indicative of greater social difficulties [40]. In line with this, task-related changes in alpha power were also observed in children diagnosed with high-functioning ASD during a motor task [66]. For those diagnosed with ASD, event-related desynchronization was observed in the occipital and parietal cortex when preparing to initiate a task related to moving the object presented but not initiating movement, defined as the ‘prepare’ stage, whereas once told to move into the ‘go’ stage the event-related desynchronization was observed in the parietal and motor cortex [66]. Neural repetition suppression to tactile stimulation (i.e. Tactile suppression index) captured by changes in alpha desynchronization with repeated stimulation, has recently been shown to be reduced in 10-month old infants with an increased familial likelihood of developing ASD relative to infants at typical likelihood of the condition [67]. This measure appeared to be a predictor for higher levels of developing ASD traits at 24 months of age [67].

Unlike that observed in the delta and theta frequencies, the evidence of a role for alterations the alpha band in ASD appears to be more consistent, albeit region-dependent, and may reflect potential for the alpha wave signature as a biomarker across ASD subtypes. The aforementioned reported correlations of alpha activity to sociality scores [40] and movement [66], for example, suggest that other behavioural symptoms on the spectrum also may be correlated to oscillatory activity in a frequency-specific manner.

### Beta

Clinical studies have identified that beta oscillatory power is reduced in frontal, temporal, parietal, and occipital brain regions [48, 63, 66, 68], with task-related changes in beta connectivity [63] and beta power [68] correlated with capabilities in number estimation and the naming of simple objects, respectively, in adults with ASD. Specifically, individuals with ASD displayed reduced inter-regional phase locking between the occipital cortex to the frontal, parietal, and temporal cortices [63], and reduced beta power [68] during a visual stimuli task and naming task respectively. Motor task-related beta changes are also observed in children diagnosed with high-functioning ASD [66]. Event-related desynchronization was observed in the beta band in the parietal cortex in the same ‘prepare’ phase, as described in the previous section, whereas during the ‘go’ stage there was event-related desynchronization in the parietal and motor cortex [66]. A reduction in beta power in the frontal cortex was also evident at rest in infants 6-months of age that had an increased familial likelihood for developing ASD [47]. Of note, however, was the finding that beta power increased over time in these children to levels higher than the control children by 24 months of age, and thereby suggesting that alterations in beta power may play a key role in ASD progression [47]. An increase in beta coherence was also observed between the inferior frontal gyrus and left fusiform gyrus [68], as well as the superior temporal gyrus in the occipital lobes of children with ASD [68, 69], and was postualted to be correlated with altered visual perception [69]. To our knowledge, there have been no preclinical studies performed evaluating a role for beta power or coherence in ASD.

### Gamma

Along with alpha, gamma oscillations have been more extensively studied in ASD, with clinical studies demonstrating higher gamma power in frontal, parietal and temporal regions in children [70] and adolescents [71]. In adults diagnosed with fragile X syndrome, elevated gamma power was reported in the frontal and occipital regions, differences that were correlated to disturbances in sensory and social processing [51, 72, 73]. It has been postulated that elevated gamma power at the midline, frontal, and parietal brain regions may reflect a possible excitatory/inhibitory imbalance within these regions, which contributes to cognitive and perceptual processes that play an important role in the behaviour expressed [29]. Reduced motor-related gamma power in children with ASD is also suggested to reflect the excitatory/inhibitory imbalance present in ASD and contribute to the behavioural profiles associated with the disorder [74]. Further, minimally verbal children (4–7 years of age) diagnosed with ASD have exhibited a reduction in gamma power within the anterior cingulate cortex while processing visual stimuli [55]. Clinical coherence studies in children showed elevated cortical gamma connectivity between the inferior frontal gyrus and the fusiform gyrus as well as between the superior temporal gyrus and occipital lobe, thought to play a role in visual processing differences in ASD [68]. As well, lower gamma coherence between bilateral posterior superior temporal sulci is thought to disturb visual integration in adults with ASD [75]. Furthermore, a decrease in gamma phase locking, which is a measurement of the consistency between instantaneous phases of oscillations and is important for communication between cortical networks, has frequently been reported in adolescents and adults with fragile X syndrome [72, 73]. This signifies a potential interference with the coordination of information within networks in individuals with fragile X syndrome and, depending on the regions involved, likely impact cognitive and behavioural processes.

Using the valproic acid (VPA) model of idiopathic ASD, one study demonstrated lower gamma connectivity in the superior temporal gyrus between hemispheres that was proposed to be correlated to social and auditory behaviours similar to that in ASD [76]. Consistent with the clinical findings in patients with fragile X syndrome [73], *fmr-1* knock out mice exhibited higher gamma power within the auditory and frontal cortex, an effect exacerbated when the animals were in motion [49]. In addition, when the gamma band was further separated into low and high frequency gamma in each region, this motion-induced increase in power was greater in high gamma range [49]. Similar to clinical findings, there also exists a decrease in gamma phase locking specifically involving fast-spiking inhibitory neurons in the somatosensory cortex of *fmr-1* knock-out mice and it was suggested to be correlated to the cognitive atypicalities present in fragile X syndrome and other ASD subtypes [77].

The identification of region-specific neurophysiological patterns associated with ASD is of vital importance to identify subtype-specific biomarkers, yet studies to date are for the most part inconsistent. We hypothesize these inconsistencies arise, at least in part, from a lack of clear identification of all of the participant symptoms, and hence identification of the ASD subtype under investigation. As a neurodevelopmental disorder, it is not surprising that age is also an important determinant of both behavioural output and neurophysiological alterations. Indeed, behavioural symptoms in ASD are not static, with manifestations in early development being lost, or even reversing, during aging [78, 79]. Therefore, more longitudinal studies are necessary to provide insights into how neuronal systems activity changes during development and into adulthood, and the relationship of these neurophysiological changes to symptom alterations. Further, many of the findings described above outlined resting-state oscillatory activity, despite the understanding that these oscillations are tightly coupled to behavioural states and that behaviours vary along the spectrum. Additional research evaluating the relationship between task-related alterations in neuronal oscillatory activity in ASD is therefore required if we are to correlate these changes to specific behavioural symtpoms.

Finally, despite an abundance of evidence that sexual dimorphisms exist in ASD symptom manifestation, little research has been performed to determine whether or not sex differences in neuronal oscillatory function contribute to these behavioural differences. Below we describe what is known to date about how males and females differ in oscillatory systems function in ASD.

## Sex differences in neuronal oscillatory function in ASD

There is a significant lack of research that identifies sex-specific differences in neurodevelopmental, neurological, and neuropsychiatric disorders, despite established sex differences in prognosis, diagnosis and symptomatology in numerous disorders. A comprehensive review by McCarthy and colleagues [80] outlined the importance of inclusion of sex differences in research and discussed a strategy for its incorporation. It is notable however that while hormones invariably play a key role in sex differences, adult sex differences may arise through developmental exposure to hormones and not necessarily by present-day changes in hormonal fluctuations and actions. It is therefore important that research including sex differences extend beyond hormones to encompass other systems so sex-specific molecular, cellular, and systems determinants of symptoms can be elucidated, and individualized treatment strategies developed.

There is evidence spanning over several decades showing that there are sex differences in neuronal oscillatory function between neurotypical men and women. For example, compared to men, women exhibit greater resting state and restfulness total EEG power [81–83] as well as while performing verbal and spatial tasks [84, 85]. In line with this, at rest and during cognitive tasks, women have been reported to have larger interhemispheric alpha coherence, indicative of a greater similarity in EEG activity between hemispheres [82, 84, 86]. Sex differences in neuronal oscillatory activity also exist for varying emotions, as shown following exposure to movie clips that induce specific emotional states, such as, disgust, fear, sadness, happiness or neutral emotional states [81]. In adults, although the expression of happiness or fear elicited similar alterations in EEG activity in both sexes, men showed increased theta, alpha and beta power in the frontal and temporal lobes during states of disgust and sadness compared to women, in addition to increased frontal alpha power during neutral emotional states [81]. These results indicate that distinct patterns of neuronal activity underly emotional expression and cognitive performance in a sex-specific manner, and are supported by preclinical studies showing sex-specific neuronal oscillatory differences in animals that are correlated to behavioural output, such as auditory fear discrimination or depression-like behaviour [87, 88]. In addition, clinical and preclinical findings have identified hormonal involvement in the regulation of neuronal oscillations under neurotypical conditions and in neuropsychiatric disorders such as major depressive disorder [30], a disorder that also demonstrates robust sex differences.

Given the tight coupling of oscillations to behavioural output, and the known sex-specific structural, functional, and behaviour differences present in the disorder [20, 37, 89], it is likely that sex differences in neuronal connectivity also exist in ASD. These neurophysiological differences remain for the most part unexplored, however some research has been performed, with a summary of the available studies that evaluated sex differences in ASD provided in Table 1. It has been reported that male adults and children with ASD predominantly express hypoconnectivity in the default mode network (DMN), a brain network active at resting state, and that such connectivity patterns are similar to that of typical females and neuronal feminization [20, 89, 90]. Conversely, females within the same age range exhibit hyperconnectivity in the DMN, which is comparable to typical male connectivity levels or neuronal masculinization [20]. It has also been shown that high-functioning female individuals with ASD between the ages of 8 and 17 present increased connectivity between the nucleus accumbens and sensorimotor brain regions that are involved in social cognitive processes, whereas their male counterparts tend to show reduced connectivity between these regions [91]. Furthermore, research on infants that have a greater chance of developing ASD from prenatal insults [92] or familial risks [93] has identified potential age-related and sex-specific alterations that could relate to the development of ASD in some instances. For example, in infants that were 3 months of age with elevated ASD likelihood no sex-specific alterations were identified, although this group showed significantly reduced beta and high-alpha total power across the frontal region for both sexes [93] that may relate to the hypoconnectivity exhibited within the DMN in males that have ASD [20, 89, 90]. However, in infants 12–36 months of age, increased EEG power in the right frontal cortex (22–24 Hz) and in the right temporal cortex (13–36 Hz) was observed in male children with an elevated familial chance of developing ASD, but not in female children from the same group [92]. Few studies have looked at regional sex differences at select oscillatory frequencies, although some evidence indicates greater oscillatory atypicalities in female children with ASD due to slow wave, focal, diffuse, and paroxysmal spike patterns observed through EEG [94], and a higher peak posterior/anterior alpha power ratio in male children [60].

**Table 1:** Research incorporating sex differences in neuronal oscillations in ASD.

Study	Diagnostic tool	Species and Age	Findings in ASD group
Brito et al., 2019 [92]	EEG	Human males and females 24–36 months compared with neonatal data	Young children with a higher familial likelihood of ASD exhibited sex and age specific neuronal correlates. Neonatal male, but not female, infants with increased ASD probability displayed increased high frequency waves within the right frontal, left parietal and right temporal lobes.
Levin et al., 2017 [93]	EEG	Human males and females 3–36 months of age	Young children with increased familial likelihood of developing ASD displayed decreased frontal power in the alpha frequency at 3 months of age, with no sex differences.
Matlis et al., 2015 [60]	EEG	Human males and females 4–8 years of age	Male children with ASD exhibited a greater alpha peak ratio compared to female children.
Tsai et al., 1981 [94]	EEG	Human, age not identified	Female individuals with ASD displayed more abnormal EEG recordings due to focal or diffuse spike, slow wave, or paroxysmal spike patterns. More females showed increased development of epilepsy.
Alearts et al., 2016 [20]	fMRI	Human males and females 7–30 years of age	Male individuals with ASD exhibited hypoconnectivity in the default mode network (DMN) while female individuals with ASD exhibited hyperconnectivity.
Hernandez et al., 2020 [91]	fMRI	Human males and females 8–17 years of age	Female adolescents with ASD, coincident with elevated oxytocin receptor gene expression, selectively displayed greater connectivity between the nucleus accumbens – subcortical regions and the prefrontal cortex.
Jung et al., 2015 [89]	fMRI	Human males mean age: 23.8 Females mean age: 22.4	In the DMN, women with ASD selectively exhibited higher fractional amplitude of low-frequency fluctuations within the posterior cingulate cortex. Men with ASD exhibited an increase in fractional amplitude of low-frequency fluctuations in the inferior frontal gyrus and the cerebellum.
Ypma et al., 2016 [90]	fMRI	Human males and females 12–18 years of age	Hypoconnectivity in the DMN in adolescent females with ASD and a trend towards hypoconnectivity in adolescent males with ASD.

There does appear to be an acknowledgement that neuronal oscillatory differences between males and females with ASD exist [20]. Indeed, it is possible that such differences are associated with the idea that greater familial risk factors and etiologic load may be required for symptomatic progression to occur in females, as the female sex appears to have potential protection in the development of behaviours associated with ASD [21]. Although, it must again be mentioned that symptom masking may be of significant relevance. While acknowledging that a handful of studies have explored sex differences in neuronal oscillations in individuals with ASD there remains a significant gap in our knowledge, from the lack of identification of frequency-specific changes, as well as to the mechanisms that may underlie any observed systems function differences. There is a critical need for this research if sex-specific oscillatory signatures are to be developed, which may aid in the prevention of misdiagnosis or delayed diagnosis, which is often seen in female children with ASD.

## The regulation of neuronal oscillations in ASD

There is currently no cure or one standard pharmacological treatment for ASD, rather the range of available treatment strategies aim to provide symptom relief. Current pharmacological therapeutics for ASD typically target behavioural aspects of the disorder and/or comorbidities, for example to improve difficulties in social and communication skills, managing irritability and attention deficits, or anxiety and depression [95–98]. It has been recently suggested that therapeutic targets for ASD should be done based on molecular alterations in the disorder [99]. Yet the identification of therapeutic targets in ASD remains limited, and is confounded by the multitude of disorders present within the spectrum, as well as the lack of incorporation of sex as an experimental variable. One potential option is to develop frequency- and region-specific oscillatory maps, that are also sex- and subtype-specific, and thus allow for more targeted treatments based on these neurophysiological biomarkers. Indeed, this idea has merit given reported links between oscillations and behaviour in ASD, such as the reduced gamma-band power in parieto-occipital regions correlated to difficulties in perceptual organization in adults with the disorder [100, 101]. Further, aberrant oscillatory patterns have been shown to be normalized by therapeutic drugs in various disorders such as schizophrenia [102, 103] and major depressive disorder [28, 104] in both clinical and preclinical studies. Thus therapeutic efficacy could potentially be determined via the degree of oscillatory normalization, and be used as an additional outcome measure alongside behaviour when testing novel therapeutics. In this regard, in the search for these novel therapies, targeting select molecular pathways known to be involved in the regulation of oscillations may be worthwhile.

Neuronal oscillations are highly regulated by various signalling pathways, for example gamma waves are highly regulated by glutamatergic excitatory and GABAergic inhibitory actions [105, 106]. Alterations in glutamatergic neurotransmission are suggested to play an underlying role in ASD as increased glutamate serum levels have been observed in children [107] and in adult males [108] with ASD. Serum glutamate levels also appear to be correlated to social behaviours and imply altered brain glutamatergic neurotransmission in these individuals [108]. Enhanced excitatory activity in ASD is also supported by work that revealed a lower GABA/glutamate metabolite concentration ratio, as GABAergic activity was decreased and glutamatergic activity increased in the occipital region in adolescents with ASD compared to controls [109]. An alteration in glutamate transmission is also evident from preclinical studies. For example, it has been shown that mice lacking the *Rab39b* gene, which is involved in the insertion of GluA2/GluA3 α-amino-3-hydroxy-5-methyl-4-isoxazolepropionic acid receptor (AMPAR) subunits at synaptic terminals, express abnormal AMPAR expression and synaptic function and is suggested to cause X-linked intellectual disability, and potentially similar cognitive deficits that occur in ASD [110]. Further, normalizing N-methyl-D-aspartate receptor (NMDAR) function in models of ASD, for example stimulating NMDAR function in the *Shank2* mouse mutant model [111] or suppressing receptor function in the VPA model of idiopathic ASD [112], has been shown to normalize social behaviours associated with ASD. Although these disparate findings likely reflect differences between the models themselves, it also raises questions as to the potential mechanistic differences that may exist across the ASD spectrum. Alterations in GABAergic neurotransmission have also been observed, with preclinical work in VPA rats showing reduced expression of GABAergic glutamic acid decarboxylase 67 (GAD67), an important enzyme for GABA biosynthesis, in the hippocampus, cerebellum, and temporal cortex and increased expression in the prefrontal cortex, changes suggested to contribute to the excitatory/inhibitory imbalance associated with the disorder [113]. As well, impairments alterations in GABAergic inhibitory transmission in the temporal cortex [114] and deficits in the *gabrb3* gene leading to the production of less efficient GABA_A_ receptors [115] have also been suggested to contribute to an imbalance in the excitation/inhibition (E/I) ratio. A dysregulation of the E/I ratio has been suggested to be linked to social difficulties in ASD [116], as well as been demonstrated to impact cortical network activity through the disruption of oscillatory phase locking [101]. Also involved with protein function, including those involved in GABA transmission, is the mechanism of S-nitrosylation (SNO), a process that plays a pivotal role in the progression of numerous disorders [117]. It was recently reported that sex differences exist between protein SNO in mice, with male- and female-specific protein SNO in the cortex, as well as sex-specific differences in the SNO levels of other proteins [117]. This may be of importance in ASD as in the *Shank3* mouse model alterations in SNO of proteins involved with neurotransmission, as well as neurodevelopment of the cortex was shown [118].

Another protein that has been linked to both cognitive dysfunction and neuronal oscillatory activity is glycogen synthase kinase-3 (GSK-3) [28]. GSK-3 is a serine/threonine kinase that acts through various intracellular pathways as a key signalling molecule in numerous biological processes [28, 119–121]. GSK-3 has key cellular roles in neurogenesis, neuronal development, learning, and memory [122] and has been recently postulated as a homeostatic regulator of neuronal oscillatory activity [28]. In preclinical studies, the overexpression of GSK-3 has been associated with reduced whole brain volume in GSK-3β[S9A] transgenic mice, mice with persistent activation of GSK-3 [123], and disruptions in learning and memory in the VPA model of ASD [124]. Increasing GSK-3 activity in the prefrontal cortex or hippocampus of rats, via adenoassociated viral infection with the GSK-3β[S9A] mutant, was also shown to directly regulate oscillatory function and induced learning and memory deficits [125]. Therefore it is not surprising that altered activation of GSK-3 in various disorders of cognitive dysfunction have been identified, such as increased activity in models of Alzheimer’s disease [126] and both an increase and decrease in activity in models used to study aspects of schizophrenia [127, 128]. A role for GSK-3 in ASD has also been proposed, with evidence coming from both clinical and preclinical studies, although findings are not in agreement as to whether GSK-3 activity is up- or downregulated [124, 129–135]. Clinically, decreased GSK-3 activity has been found in T cells from children with ASD, potentially related to an elevation in the Akt/mammalian target of rapamycin (mTOR) pathway, an upstream regulator of GSK-3 [133]. At a preclinical level using genetic models of ASD, GSK-3 activity has been shown to be downregulated in the anterior cingulate cortex of *shank3b* knock-out mice [135], whereas its activity is upregulated in the striatum, cortex, and the hippocampus of *fmr-1* knock-out mice [131]. In the idiopathic VPA model of ASD, GSK-3 activity is suppressed in several regions that include the cerebellum, prefrontal cortex, hippocampus, and amygdala, that is also associated with increased levels of β-catenin [124, 134]. Activation of the GSK-3-βcatenin pathway was also shown to underlie macrocephaly in VPA animals [136], a phenomenon also observed in individuals with ASD [137, 138]. In the VPA model, there was also decreased levels of phosphatase and tensin homolog (PTEN) in the hippocampus and cortex, a protein involved in mitochondrial biogenesis through the AKT/GSK-3β/PGC-1α signaling pathway, and which led to increased GSK-3 phosphorylation and reduced activity [139]. Interestingly, a higher level of GSK-3 phosphorylation was also found in the amygdala of VPA rats as a result of attenuated NMDAR-induced synaptic plasticity [124], which could also play a key role in oscillatory activity. Further to these findings, selective and non-selective GSK-3 inhibitors improved cognitive performance in both clinical [129] and preclinical [140–142] fragile X syndrome studies, which supports the use of GSK-3 inhibitors, such as lithium, as therapeutics for ASD. Together these findings suggest that GSK-3 may play a central role in ASD pathology, although whether it is up- or downregulated appears to be region- and model-dependent. It is therefore an interesting notion that GSK-3 may have differing involvement in ASD that may be dependent upon the subtype under investigation. Nonetheless, if the hypothesis regarding GSK-3 as a homeostatic regulator of neuronal oscillations [28] holds true, the therapeutic regulation of this kinase may be a valued approach for several of ASD disorders.

## Conclusion

Determining biomarkers for the underlying mechanisms contributing to the various disorders across the ASD spectrum could further advance diagnosis as well as identify potential therapeutic targets for this group of disorders. In the case of neuronal oscillations, normalization of aberrant patterns may represent a valued approach to study novel therapeutics, as alpha and gamma oscillations have already been suggested as potential biomarkers of ASD [76, 143]. It is worth noting, that studies to date have grouped the ASD subtypes together, and therefore, frequency-specific changes within specific subtypes of the disorder have not been thoroughly identified. Further to this, adding sex differences as an additional readout of these measures is required, especially given the widely reported differences in prevalence, age of onset, and symptoms between male and female individuals with ASD. It would be of significant value to therefore generate neurophysiological maps that are not only sex-specific, but also, that have incorporated regional changes based on ASD subtype.

This review identifies the urgent need for more clinical and preclinical research combining sex differences in the pathophysiology of ASD with novel strategies to identify biomarkers and therapeutics for all disorders on the spectrum. Given the varying symptoms and profiles of ASD it is unlikely that a one-drug-one-target strategy will be successful, and it is therefore necessary to be diligent in our biological assessments of individuals with ASD if we are to advance future prognosis and an ASD diagnosis in a timely manner. With no present pharmacological treatment options, a more stringent evaluation of the molecular underpinnings through the utilization of these discrete measures may allow for sex- and subtype-specific symptoms of social behaviour, intellectual disability and visual and/or auditory alterations to be alleviated.
